# Computerized Morphometric Analysis of Human Femoral Articular Cartilage

**DOI:** 10.5402/2012/360201

**Published:** 2012-02-15

**Authors:** Neeru Goyal, Madhur Gupta

**Affiliations:** ^1^Department of Anatomy, Health Sciences Block, Christian Medical College, Ludhiana 141008, India; ^2^Department of Anatomy, Swami Devi Dayal Hospital & Dental College & Hospital, Golpura 134118, India

## Abstract

*Objective*. Articular cartilage shows changes with age that are considered to be the most important factors in the development and progression of osteoarthritis. The studies on age changes in articular cartilage have been traditionally based on individual observations but this approach is limited by its subjectivity and bias, yielding considerable variability. So the present study was conducted to observe various age related changes in morphology of femoral articular cartilage using computerized morphometric analysis. *Design*. The articular cartilage specimens were divided into two groups according to age: group 1 (*n* = 16) below 40 years (16–40 years) and group 2 (*n* = 12) above 40 years (41–86 years) of age. 5 *μ*m thick paraffin sections were stained with H&E and analyzed using Image Pro Express image analysis software for quantitative analysis of articular cartilage. Various parameters, that is, total thickness of the cartilage, area of lacunae in each zone, area of subchondral cavities, and number of chondrocytes per 10,000 *μ*m^2^ area in each zone were measured. *Results*. Significant difference with age was found in the total thickness and area of lacunae in zone 3. *Conclusions*. Not much difference is observed in articular cartilage morphology with age. So ageing is not the only risk factor in development of osteoarthritis.

## 1. Introduction

Articular cartilage is a specialized connective tissue that covers the ends of the bones in synovial joints. Articular cartilage extracellular matrix and cell function change with age and are considered to be the most important factors in the development and progression of osteoarthritis. Aging and development of cartilage degeneration involve many factors, which either alone or in combination may precipitate the onset of osteoarthritis. Much evidence indicates that a single factor may induce a number of sequential responses and structural changes, which either affects the extracellular matrix or cell function, or makes the tissue more vulnerable to compressive loads or injury. These changes eventually lead to a disruption of tissue homeostasis and reduced capacity for regeneration, which manifest as osteoarthritis and eventual tissue destruction. The aging process is inevitable, yet our understanding of the consequences of this phenomenon is incomplete [1]. The relationship between aging and osteoarthritis is clinically and epidemiologically evident. Articular cartilage appears to be highly susceptible to the accumulation of aging-related changes, in part due to the relatively low turnover of extracellular matrix and cells. It has been estimated that the half-life of type II collagen is approximately 117 years and various measurements in mature cartilage revealed only a very small fraction of proliferating cells. Thus, cartilage matrix and cells are prone to accumulate changes related to trauma, mechanical, or oxidant stress over time [2].

The studies on age changes in the articular cartilage have been traditionally based on individual observations but this approach is limited by its subjectivity and bias, yielding considerable variability. In this study, we used Image Pro Express image analysis software for quantitative analysis of articular cartilage. Computerized morphometric analysis provides a simple, reliable, and reproducible method for improved and consistent results in various research works. So the present study was conducted to observe the various age related changes in the morphology of the femoral articular cartilage using computerized morphometric analysis.

## 2. Materials and Methods

The articular cartilage specimens were divided into two groups according to their age.

**Group 1 (*n* = 16)**

Below 40 years (16–40 years) of age as the articular cartilage below 40 years showed a normal adult histology and

**Group 2 (*n* = 12)**

Above 40 years (41–86 years) of age.

Patients suffering from any disease of knee joint like tumor, any deformity, bony enlargement, or irregularities such as osteophytes or having a history of trauma or joint pain were excluded from the study.

Articular cartilage specimens were obtained from the most prominent area of the medial femoral condyle from cadavers in the department of Anatomy within 18 hrs after death and from patients undergoing lower limb amputation due to accident in the Trauma center of Postgraduate Institute of Medical Education and Research, Chandigarh.

5 *μ* thick serial paraffin sections were stained with haematoxylin and eosin [3] to confirm the histological structure of the articular cartilage and to study the various features for morphometry.

 The equipment for morphometric analysis consisted of an Olympus microscope fitted with a digital camera and computer loaded with Image Pro Express software. In all specimens, every sixth section was observed. At least five sections of each specimen and five fields from each section were observed and photomicrographed. The image acquired on the computer was analyzed using the Image Pro Express software for the following.

- Total thickness of the articular cartilage.
- Area of lacunae in each zone.
- Area of subchondral cavities.
- Number of chondrocytes per 10,000 *μ*m^2^ area in each zone.

For “*total thickness*” of the articular cartilage, sections were photomicrographed at 4X magnification, the software was calibrated for 4X magnification, and thickness was measured in millimeters using “measure the distance” option of the software.

For “*area of lacunae and subchondral cavities*” sections were photomicrographed at 40X magnification. The software was calibrated for 40X magnification and the area was measured in square microns by drawing the area to be measured using the “area of interest (AOI)” option of the software. Area of minimum of 1000 cells was measured for every specimen.

 For measuring the “*number of chondrocytes per 10,000  μ*m^2^,” photomicrographs at 40X magnification were used. The software was calibrated for 40X magnification and the area was measured in square microns by selecting the area. Number of cells in that given area was counted using the “tag points” option of the software.

## 3. Statistical Analysis

SPSS software package was used for statistical analysis. Data was expressed as mean ± standard deviation. Independent sample *t*-test (Student's *t*-test) was used to determine the statistical significance between the means. Analysis of variance (ANOVA) was done followed by Student-Newman-Keul's post-hoc tests. Partial correlation of various parameters with age was computed using Pearson's correlation test. For all statistical tests, probability levels of less than or equal to 5% (two-tailed *P* value <0.05) were considered to be significant.

## 4. Results

**Group 1**

In group 1, the articular cartilage was obtained from knee joints of individuals below 40 yrs of age as the articular cartilage below 40 years showed a normal adult histology and no significant difference was observed in the various parameters in all age groups below 40 years of age. The articular surface appeared to be very smooth without any surface irregularities. There was marked difference in the size, shape, and arrangement of the cells from superficial to deep part of the cartilage. The cells varied in shape from quite flat near the surface to oval or rounded in the deeper layers. Total thickness of the articular cartilage ranged from 2.01 to 2.89 mm with an average of  2.34 ± 0.30 mm (Table 1).

The area of the lacunae and density of cells that is, number of cells per 10,000 *μ*m^2^ area in the four zones of the articular cartilage were measured (Tables 2 and 3). The lacunae were classified as small sized (<100 *μ*m^2^), medium-sized (100–200 *μ*m^2^), and large sized (>200 *μ*m^2^) according to area.

1. Superficial/Tangential Zone (Zone 1). In the superficial zone, the area of the lacunae ranged from 29.62 to 107.38 *μ*m^2^ with an average of 54.93 ± 22.54 *μ*m^2^. 86.36% of the lacunae were small sized (<100 *μ*m^2^) in area and 13.64% were medium sized (100–200 *μ*m^2^). None of the lacunae were large sized (>200 *μ*m^2^). The density of cells, that is, number of cells per 10,000 *μ*m^2^ area ranged from 1.49 to 14.99 with an average of  5.76 ± 3.85.
2. Intermediate/Transitional Zone (Zone 2). In the intermediate zone, the area of the lacunae ranged from 109.78–255.66 *μ*m^2^ with an average of  154.62 ± 36.39 *μ*m^2^. 21.96% of the lacunae were small sized, 64.59% were medium sized, and 13.55% of the lacunae were large sized. The density of cells varied from 0.57 to 6.42 with an average of  3.12 ± 1.71.
3. Radiate/Deep Zone (Zone 3). In the radiate zone, the area of the lacunae ranged from 128.09 to 194.09 *μ*m^2^ with an average of  161.63 ± 17.42 *μ*m^2^. 16.46% of the lacunae were small-sized, 62.42% were medium sized, and 20.12% of the lacunae were large sized. The density of cells ranged from 1.39 to 5.31 with an average of  2.69 ± 1.12.
4. Deepest/Calcified Zone (Zone 4). The area of the lacunae ranged from 102.24 − 192.05 *μ*m^2^with an average of  145.00 ± 32.17 *μ*m^2^. 21.54% of the lacunae were small-sized, 64.62% were medium-sized and 13.84% of the lacunae were large-sized. The density of cells ranged from 0.91 − 5.82 with an average of  3.36 ± 1.37.

**Subchondral Cavities**

Deep to the articular cartilage was present the subchondral bone. Numerous small cavities were seen in the subchondral bone. The area of the subchondral cavities varied from 1,395.17 to 21,366.52 *μ*m^2^ with an average of  5933.46 ± 5821.12 *μ*m^2^ (Table 4).

Comparison of the mean of the area of lacunae and the cell density in the four zones and the mean of the area of subchondral cavities at various age groups using ANOVA showed no statistically significant difference and no correlation of these parameters with age was observed in group 1.

### 4.1. Group 2 (above 40 Years; *n* = 12)

In group 2, normal histology was seen, that is, four zones could be identified in all specimens though changes were observed in the superficial zone either in the form of horizontal or oblique splitting of collagen bundles or complete loss of the most superficial zone. The articular surface had become irregular in 9 (75%) specimens. In 6 (60%) specimens small oblique or vertical splits were present at the surface while in 1 (8.33%) specimens, the superficial zone was swollen due to separation of the collagen bundles and in 2 (16.67%) specimens, the articular surface had depressed areas giving a saucer-like appearance.

Total thickness of the articular cartilage ranged from 1.57 to 2.79 mm with an average of  2.23 ± 0.36 mm (Table 1). On comparing the mean of the total thickness at various age groups using ANOVA, no statistically significant difference in the mean of the total thickness above 40 years of age was observed. The total thickness of the cartilage in group 2 was significantly correlated to age (*P* < 0.05). The total thickness was found to decrease with increasing age.

The area of the lacunae (Table 2) and density of cells (Table 3) was measured and the lacunae were classified according to area.

The articular cartilage was observed to be consisting of four zones.

1. Superficial/Tangential Zone (Zone 1). The chondrocytes were small and flattened having oval nucleus. The area of the lacunae ranged from 35.69 to 83.70 *μ*m^2^ with an average of  58.92 ± 18.03 *μ*m^2^. 85.79% of the lacunae were small sized (<100 *μ*m^2^) in area and 14.21% were medium sized (100–200 *μ*m^2^). None of the lacunae were large sized (>200 *μ*m^2^). The density of cells, that is, number of cells per 10,000 *μ*m^2^ area ranged from 2.35 to 11.44 with an average of  6.08 ± 2.97.
2. Intermediate/Transitional Zone (Zone 2). The area of the lacunae ranged from 131.91 to228.79 *μ*m^2^ with an average of  167.79 ± 27.18 *μ*m^2^. 9.63% of the lacunae were small sized, 63.76% were medium sized, and 26.61% of the lacunae were large sized. The density of cells ranged from 1.31 to 5.93 with an average of  3.01 ± 1.35.
3. Radiate/Deep Zone (Zone 3). The area of the lacunae ranged from 150.33–300.97 *μ*m^2^ with an average of  204.59 ± 43.28 *μ*m^2^. 3.35% of the lacunae were small sized, 60.59% were medium sized, and 36.06% of the lacunae were large-sized. The density of cells ranged from 1.51 to 4.94 with an average of  3.24 ± 1.11.
4. Deepest/Calcified Zone (Zone 4). The area of the lacunae ranged from 102.24 to 192.05 *μ*m^2^ with an average of  145.00 ± 32.17 *μ*m^2^. 21.54% of the lacunae were small sized, 64.62% were medium sized, and 13.84% of the lacunae were large sized. The density of cells ranged from 0.91 to 5.82 with an average of  3.36 ± 1.37.

**Subchondral Cavities**

Deep to the articular cartilage was present the subchondral bone. Numerous small cavities were seen in the subchondral bone. The area of the subchondral cavities varied from 2,191.57 to 10,309.88 *μ*m^2^ with an average of  4322.89 ± 2299.21 *μ*m^2^ (Table 4).

Comparison of the mean of the area of lacunae and the cell density in the four zones and the mean of the area of subchondral cavities at various age groups using ANOVA showed no statistically significant difference in the various age groups above 40 years of age and no correlation of these parameters with age was observed in group 2.

### 4.2. Comparison of Groups 1 and 2

On comparing the means using independent sample *t*-test (Student's *t*-test), the mean of the lacunar size in zone 3 was found to differ significantly in the below 40 (group 1) and above 40 (group 2) age groups (*P* < 0.01). Lacunar size was found to be significantly larger in the latter (Table 5).

The total thickness and the lacunar size in zone 3 were found to correlate significantly with age (*P* < 0.05). The total thickness was found to decrease with age while the lacunar size was found to increase with age.

## 5. Discussion

The articular cartilage below 40 years of age showed a smooth surface and a distinct zonation into four zones while in group 2 (above 40 years), although zonation could be identified in all the specimens, changes were seen in the form of small splits or swelling due to separation of collagen bundles only in the superficial zone of the cartilage. The superficial zone of the articular cartilage plays a vital role in the healthy joint by virtue of its unique location, structure, and material properties. It provides a smooth surface with low friction that withstands high tensile stresses and distributes the load over the surface of the joint, thereby protecting the underlying cartilage. Disruption of the superficial zone may cause release of metalloproteinases from chondrocytes or from synovial fluid and may lead to the enhanced removal of proteoglycans from the tissue hence leading to further degeneration [4]. In previous studies on osteoarthritic articular cartilage, we observed a disrupted surface in the osteoarthritic articular cartilage.

Inspite of the changes in the superficial zone, the deeper zones appeared normal with a regular tidemark. The changes in the superficial layer which appear due to splitting of collagen bundles have been linked with a weakened or altered fibrillar network [5] and could deprive the adjacent cartilage of its nutrition leading to further changes. In previous studies, we observed an irregular articular surface and altered histology throughout the thickness of the cartilage in osteoarthritic articular cartilage [6, 7]. Wigley [8] also stated that articular cartilage has no nerves or blood vessels and its nutrition depends on peripheral vascular plexus in synovial membrane (*circulus vasculosus articuli*), synovial fluid, and blood vessels in adjacent marrow spaces though the relative importance of these is uncertain. The changes of articular cartilage with advancing age could be because of lack of its nutrition as there is an imbalance between the chondrocytes and the cartilage matrix.

The average thickness of the articular cartilage was found to be  2.34 ± 0.30 mm in the subjects below 40 years of age. Wigley [8] stated that the thickness of the articular cartilage ranges from 1 to 2 mm and may even reach 5–7 mm. Articular cartilage thickness has been extensively studied in the knee [9–12]. However, most of these studies are based on radiographic imaging, specifically magnetic resonance imaging (MRI). According to Eckstein et al. [10] MRI of strongly curved surfaces can overestimate cartilage thickness without appropriate derivations.

In the present study, the total thickness of the cartilage was found to decrease significantly with age in group 2. Based on MRI studies, Karvonen et al. [13] reported significant cartilage thinning with age in the weight-bearing aspect of the femoral condyles of subjects without osteoarthritis. Hudelmaier et al. [14] in their MRI studies found significant reduction of cartilage thickness in the femur of elderly men and women and stated that thinning of knee joint cartilage occurs physiologically with ageing, even in the absence of cartilage disease. However, this reduction is higher in some cartilages than in others. Earlier, Bullough and Jagannath [15] stated that the calcification of the cartilage is uninterrupted throughout the adult life and the calcified zone remains at the constant thickness, due to the absorption of the calcified cartilage into the subchondral bone by endochondral ossification while the calcification front (tidemark) continues to advance into the noncalcified cartilage at a slow rate which is in equilibrium with the rate of absorption of the calcified cartilage from the subchondral bone.

Comparison of the mean of the total thickness, area of lacunae, and the cell density in the four zones and the mean of the area of subchondral cavities at various age groups using ANOVA showed no statistically significant difference and no correlation of these parameters with age was observed while the lacunar size in zone 3 was found to be significantly larger in the ageing articular cartilage suggesting the cells may undergo hypertrophy.

The usefulness of the earlier methods of micrometry was inherently limited by observer subjectivity and bias, yielding considerable variability. Because of the development of digital microscopic image analysis, substantial efforts have been made to correlate the evaluations made by experienced pathologists with quantitative values [16–18]. In the present study we used computerized morphometric analysis to observe the changes in the ageing articular cartilage. Further insights in this field are required to understand the phenomenon of ageing and cartilage degeneration.

## Figures and Tables

**Table 1:** Mean and standard deviation of the total thickness of articular cartilage.

Age group	16–20 yrs	21–30 yrs	31–40 yrs	41–50 yrs	51–60 yrs	61–70 yrs	71–80 yrs	81–90 yrs
Total thickness (in mm)	2.34 ± 0.47	2.35 ± 0.28	2.29 ± 0.36	2.39 ± 0.49	2.42 ± 0.25	2.37 ± 0.21	2.41 ± 0.54	1.90 ± 0.23

**Table 2:** Mean and standard deviation of the area of the lacunae in *μ*m^2^.

	Zone 1	Zone 2	Zone 3	Zone 4
16–20 yrs	85.99 ± 0.49	165.38 ± 13.89	170.67 ± 5.77	151.52 ± 19.17
21–30 yrs	46.64 ± 12.63	157.79 ± 45.29	157.97 ± 17.62	160.30 ± 34.99
31–40 yrs	60.12 ± 33.95	141.30 ± 7.47	166.24 ± 21.16	174.93 ± 49.48
41–50 yrs	55.73 ± 2.80	156.11 ± 34.22	200.65 ± 4.46	131.57 ± 12.51
51–60 yrs	77.75 ± 8.42	147.07 ± 14.37	218.44 ± 36.18	159.06 ± 38.98
61–70 yrs	35.99 ± 0.42	204.60 ± 34.21	225.65 ± 106.52	160.42 ± 44.23
71–80 yrs	61.50 ± 30.35	189.73 ± 4.77	198.14 ± 14.33	105.49 ± 4.59
81–90 yrs	61.28 ± 15.90	154.63 ± 4.38	192.35 ± 43.75	156.75 ± 30.69

**Table 3:** Mean and standard deviation of the cell density (number of cells per 10,000 *μ*m^2^ area) in all four zones.

	Zone 1	Zone 2	Zone 3	Zone 4
16–20 yrs	3.66 ± 2.35	2.56 ± 1.88	2.82 ± 1.11	3.62 ± 1.65
21–30 yrs	6.97 ± 4.34	3.20 ± 1.65	2.83 ± 1.35	2.74 ± 1.23
31–40 yrs	3.79 ± 1.59	3.19 ± 2.24	2.29 ± 0.26	1.94 ± 0.15
41–50 yrs	5.99 ± 3.29	2.34 ± 0.70	3.14 ± 0.45	3.57 ± 1.45
51–60 yrs	9.03 ± 3.41	4.36 ± 2.22	4.28 ± 0.94	4.03 ± 0.56
61–70 yrs	9.29 ± 0.71	3.11 ± 0.50	3.76 ± 1.27	4.95 ± 1.23
71–80 yrs	3.05 ± 0.47	1.63 ± 0.45	2.00 ± 0.33	1.86 ± 0.35
81–90 yrs	4.56 ± 1.63	3.31 ± 1.34	3.13 ± 1.29	2.88 ± 1.32

**Table 4:** Mean and standard deviation of the area of subchondral cavities in *μ*m^2^ in different age group.

16–20 yrs	3618.23 ± 1865.43
21–30 yrs	6608.19 ± 7312.13
31–40 yrs	5404.25 ± 1549.55
41–50 yrs	3845.25 ± 1227.42
51–60 yrs	8419.52 ± 2673.38
61–70 yrs	2885.58 ± 981.48
71–80 yrs	3480.94 ± 1702.97
81–90 yrs	3653.03 ± 1192.75

**Table 5:** Comparison of various parameters in groupa 1 and 2.

	Group 1	Group 2	*T* value	Significance
TT	2.34 ± 0.30	2.23 ± 0.25	1.95	NS
A1	54.93 ± 22.54	58.92 ± 18.03	0.504	NS
A2	154.62 ± 36.39	167.79 ± 27.18	1.052	NS
A3	161.63 ± 17.42	204.59 ± 43.28	3.617	Significant
A4	162.86 ± 36.19	145.00 ± 32.17	1.354	NS
D1	5.76 ± 3.85	6.08 ± 2.97	0.238	NS
D2	3.12 ± 1.71	3.01 ± 1.35	0.186	NS
D3	2.69 ± 1.12	3.24 ± 1.11	1.272	NS
D4	2.65 ± 1.17	3.36 ± 1.37	1.482	NS
ASC	5933.46 ± 5821.12	4322.89 ± 2299.21	0.904	NS

*P* value of less than or equal to 0.05 has been considered significant, otherwise nonsignificant (NS).

TT, total thickness of the cartilage; A1, A2, A3, A4, area of lacunae in zone 1–4; D1, D2, D3, D4, number of chondrocytes per 10,000 *μ* ^2^ area zone 1–4; ASC, area of subchondral cavities.
